# Disease progression but not physical state per se determines mental wellbeing in ALS

**DOI:** 10.1007/s00415-020-10027-x

**Published:** 2020-07-08

**Authors:** Cynthia R. Vázquez Medrano, Helena E. A. Aho-Özhan, Ulrike Weiland, Ingo Uttner, Albert C. Ludolph, Dorothée Lulé

**Affiliations:** Department of Neurology, University of Ulm, Oberer Eselsberg 45, 89081 Ulm, Germany

**Keywords:** ALS, Wellbeing, Quality of life, Depressiveness, Progression rate, Physical function

## Abstract

**Background:**

Studies provide inconclusive results on the question whether loss of mental wellbeing is actually associated with decline in physical function in amyotrophic lateral sclerosis (ALS). The purpose of this study was to determine predictors of mental wellbeing in ALS.

**Methods:**

In total, *n* = 330 ALS patients were interviewed on parameters of mental wellbeing to evaluate the patients’ capacity of psychosocial adaptation. These parameters were global and subjective quality of life (QoL), and depressiveness. A subsample of *n* = 82 ALS patients were interviewed again within approximately a year (mean 14.34 ± 5.53 months).

**Results:**

Both global and subjective QoL were stable, whereas depressiveness increased within the course of 1 year after diagnosis. Physical function decline was associated with mental wellbeing. Progression of physical disabilities and symptom duration were significant predictors of wellbeing in the sense that fast progression and short time since symptom onset (both indicating short time to adapt) were associated with low wellbeing.

**Conclusions:**

There is evidence for subsamples in ALS with regard to mental wellbeing, which are mainly determined by clinical parameters. Those subjects being reported in the literature to present with high mental wellbeing are often long survivors. High progression rate and low physical function when attending the clinic for the first time should be red flags and need special attention in clinical counseling.

## Introduction

Amyotrophic lateral sclerosis (ALS) is a fatal diagnosis, leaving the patient in a devastating state of physical immobility and incapacity to speak [1]. Considering that there is still no cure for ALS, maintaining the wellbeing of these patients is of utmost importance and the primary goal of most therapeutic interventions available for ALS. The term “wellbeing” can include many aspects such as the physical or mental state, or social life of a person. Thus, in ALS mental wellbeing might be good despite severe physical impairments. For healthy subjects, this might be hard to understand as they preclude from their own fears and expectations, and thus significantly underestimate ALS patients’ wellbeing [2].

Since the early 1990s, there have been reports of satisfactory mental wellbeing despite the severe loss of physical function in ALS, and that mental wellbeing depends on factors other than clinical parameters [3, 4]. Indicators of mental wellbeing are low depressiveness and high levels of quality of life (QoL), both parameters provide evidence for successful psychosocial adaptation. Findings of good mental wellbeing in ALS have been challenged by other studies proclaiming positive correlation between the progression of physical disabilities and mental wellbeing, indicated by increase in depressiveness and loss of QoL, e.g., Shamshiri et al. suggested that during the course of 1 year, QoL decreases as the physical and functional abilities deteriorate [5]. Others suggest that depressive symptoms may occur as a reaction following the communication of ALS diagnosis [6], or that depression might be present even 1 year before symptom onset already [7]. Lulé et al. concluded that progression rate might significantly determine patients’ wellbeing in the sense that fast progressing patients had few chances to adapt [2], which was further supported by Körner et al., who provided evidence of this within the first 2 years after diagnosis [8]. Very fast rates of disease progression leave less time for patients to adapt to the situation. In case the disease progresses slowly, patients have more time to adapt and may manage to maintain a satisfactory QoL and low levels of depressiveness, making time a key issue of psychosocial adaptation to the disease.

Other studies suggest that depressiveness is not necessarily expected in ALS patients and that a good QoL can be maintained even in the late stages of the illness or in locked-in syndrome patients [9–11]. In a longitudinal study of 2 years by Matuz et al. [12], results indicated that depressive symptoms and QoL can be stable during the course of the disease, if patients have sufficient resources to cope with the situation. Similar results can also be found in other studies, which confirm a stable psychosocial adjustment in the course of the disease [2, 13, 14].

One major challenge is the heterogeneity of study samples, which might explain some of the variance between studies. Further, there are only few longitudinal studies with a large clinical cohort of patients, but instead, highly selected subsamples of (possibly) long surviving patients are reported within some studies and challenged by other studies with patients shortly after diagnosis. We hereby present data from a large clinical sample followed over the course of 1 year to disentangle those dynamics of psychosocial adaptation in association with clinical characteristics of patients.

## Methods

### Subjects

In a cross-sectional study, *n* = 330 patients (mean age 61.19 ± 11.69 years) with the diagnosis of ALS, according to revised El Escorial criteria [15], who visited the in- or outpatient clinic of the University of Ulm between August 2012 and June 2016, were included in the study. Patients had an average of 34.17 ± 38.66 months since symptom onset. Exclusion criteria were neurological illness other than ALS, including the clinical diagnosis of amyotrophic lateral sclerosis and frontotemporal dementia (ALS-FTD) according to Strong criteria [16], or poor knowledge of the German language.

For follow-up, patients were interviewed after approximately 1 year (mean 14.34 ± 5.53 months). In total, *n* = 82 of the patient sample were included for follow-up interviews (Table 1). Dropouts for follow-up were due to death (*n* = 70), not attending the clinic a second time (*n* = 114) and physical or mental inability (*n* = 17). Forty-seven patients declined to do a second interview, within this group of dropout patients; there was no evidence of increased depressiveness compared to the participating patients.

**Table 1:** Demographics and clinical data

	First interview (*n* = 330)			Second interview (*n* = 82)		
	M	SD	Range	M	SD	Range
Gender	132f/198 m			26f/56 m		
Age	61.19	11.69	19–84	61.82	11.75	32–83
Symptom duration (months)	34.17	38.66	3–396	59.64	58.92	13–417
Time since diagnosis (months)	13.25	19.54	0–144	29.90	21.85	9–126
Spinal/Bulbar	246/84			61/21		
Sporadic/Familial	306/20^a^			77/4^b^		
Physical disability (ALSFRS-r)	35.23	8.20	6–48	29.00	10.15	5–45
Non-invasive/invasive ventilation	99/1			38/0		
PEG	28			11		
Progression rate	.64	.60	.00–4.86	.52	.41	.02–2.63

*SD* standard deviation, *ALSFRS-r* ALS functional rating scale-revised, *PEG* percutaneous endoscopic gastrostomy

^a^Information on four patients not available

^b^Information on one patient not available

Physical disability was assessed by the revised ALS Functional Rating Scale (ALSFRS-r), with a maximum score of 48 indicating normal physical function [17]. Progression rate was calculated by subtracting the individual ALSFRS-r score from the maximum ALSFRS-r score, and calculating the ratio of this difference and the time since onset [18]. A rapid loss of physical functions are more than 1.4 points per month according to Lulé et al. [2].

Patients were interviewed on their subjective psychological wellbeing, measured by depressiveness and QoL. Depressiveness was measured with the ALS depression inventory—twelve items (ADI-12) [19]; a total score ≥ 23 indicates symptoms of minor depressive disorder and ≥ 29 indicates symptoms of clinically relevant depression. Global QoL was assessed with the Anamnestic Comparative Self Assessment (ACSA) [20], with a range from as bad as possible (-5) to as good as possible (+ 5). For subjective QoL, we used the Schedule for the Evaluation of Subjective Quality of Life (SEIQoL) [21]. For SEIQoL, patients name five areas of their life that are most important for their overall QoL and then they rate how satisfied they are with these areas at the moment. A SEIQoL index score can be determined within a range from 0 to 100 [21]; > 70 indicating good QoL.

The interviews lasted about 1 hour and were part of a larger study on therapeutic decision-making published elsewhere (www.NEEDSinALS.com) [22].

### Statistical analyses

Statistical analyses were performed using the Statistical Package for Social Sciences (SPSS, IBM, version 21.0). Mean values and standard deviations are given in the tables. Normality of the data was tested with the Kolmogorov–Smirnov test and either parametric or non-parametric statistical tests were applied accordingly.

Spearman correlation was applied for the association of wellbeing (depressiveness, QoL) and clinical data (physical disability, progression, symptom duration and time since diagnosis). A linear regression analysis, including best curve fitting, was conducted with the variables of wellbeing and clinical data.

In the longitudinal analysis, a pairwise t-test and a Wilcoxon signed-rank test were performed to compare the scores of depressiveness and QoL of the first and the second assessment. The threshold for significance was set at *p* < 0.05.

## Results

### Initial state of wellbeing

In the first interview, the global (ACSA) and the subjective (SEIQoL) QoL indicated an overall satisfactory mean of QoL in the patient sample. The mean depression score, measured with ADI-12, showed clinically relevant depressive symptoms in 19% (*n* = 60) of patients (Table 2).

**Table 2:** Indicators of wellbeing

Measures of wellbeing	First interview		Second interview		
	*n* = 330		*n* = 82		
	M	SD	M	SD	*p*
Physical disability (ALSFRS-r)	35.23	8.20	29.00	10.15	.000*
Global QoL (ACSA)	.15	2.54	.22	2.43	.970
Subjective QoL (SEIQoL)	73.26	15.89	74.93	13.92	.146
Depressiveness (ADI-12)	23.42	6.63	23.81	7.03	.046*

*ALSFRS-r* ALS functional rating scale-revised, *ADI-12* ALS depression inventory—twelve items, *ACSA* anamnestic comparative self-assessment, *SEIQoL* schedule for the evaluation of subjective quality of life

Wilcoxon signed-rank test

*Significance with the level of *p* < 0.05

### State of wellbeing after 12 months

Over a period of approximately 12 months, there was a significant decline in the mean score of physical function (*p* < 0.01), losing 6.23 points on average on the ALSFRS-r scale. Mean global QoL at second interview was 0.22 and mean subjective QoL was 74.93%, indicating that patients presented no significant change in global (*p* = 0.970) or subjective QoL (*p* = 0.146) within a period of 12 months (Table 2).

An increase in the ADI-12 score by 0.39 points indicated a significant rise in depressiveness (*p* = 0.046) between the first and the second interview. Clinically relevant depressiveness on the second interview was observed in 22% (*n* = 18) of patients.

### Association of clinical parameters and wellbeing

**Fig. 1:**
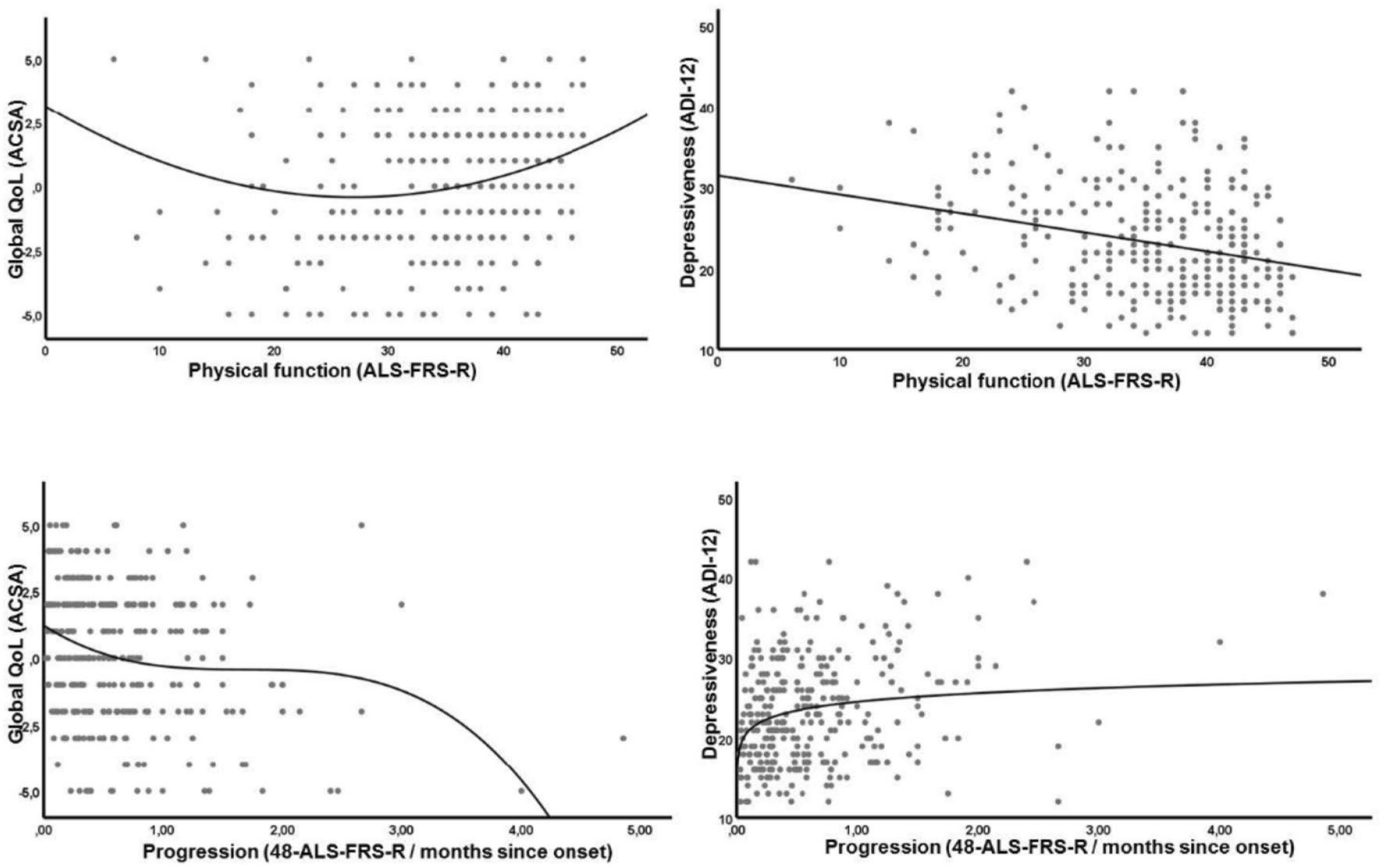
Association of indicators of wellbeing (global QoL indicated by ACSA, depressiveness indicated by ADI-12) and clinical parameters (physical function indicated by ALSFRS-r score and disease progression indicated by the average loss of physical function measured with ALSFRS-r per month). Regression analysis with curve fitting; always the best fit is displayed. Threshold of significance was set at *p* < 0.01

Regression analysis revealed no significant association between time since onset or time since diagnosis and any measures of wellbeing. A significant relation was seen for physical functions and some measures of wellbeing: significant quadratic curve fitting for global QoL (QoL declines as physical function decreases but in advanced disease states, QoL is high again) and linear fitting for depressiveness (general increase in depressiveness, the more physically impaired the patients are; Table 3, Fig. 1).

**Table 3:** Association of clinical parameters and wellbeing

Clinical data		Measures of wellbeing		
		Global QoL (ACSA)	Subjective QoL (SEIQoL)	Depressiveness (ADI-12)
Physical function (ALSFRS-r)	Curve fitting	Quadratic	No relation	Linear
	*R*^*2*^	.046		.073
	*p*	.031*		.000*
Disease progression (48—ALSFRS-r/time since onset)	Curve fitting	Cubic	Cubic	Logarithmic
	*R*^*2*^	.058	.025	.040
	*p*	.036*	.041*	.034*

Regression analysis for the association of clinical parameters and wellbeing with curve fitting; always the best fit is displayed. There was no significant fit for time since onset or time since diagnosis and any measure of wellbeing

*Significance with the level of *p* < 0.05

Progression of the disease was associated with all measures of wellbeing (global and subjective QoL, and depressiveness; Table 3, Fig. 1). Disease progression and both global and subjective QoL revealed a cubic fitting, indicating a significant drop in QoL, the faster the disease progresses (turning point of the curve at progression rate of 3 or more, indicating a loss of physical function according to the ALSFRS-r score of more than 3 points per month). Disease progression and depressiveness showed a logarithmic curve fitting, indicating that depressive state significantly increased the faster the progression of the disease. There were no significant changes in measures of wellbeing related to time since diagnosis or time since disease onset (data not shown).

### Subgroup analyses

Further, patients were divided into subgroups according to progression rate for “fast” (*n* = 28) and “slow progressors” (*n* = 301) (≥ 1.4 and < 1.4 loss of points on the ALSFRS-r per month, respectively; Table 4) [2]. Only patients with a fast disease progression showed a significant increase in depressiveness the more advanced their physical disability (*R*^*2*^ = 0.056, *p* = 0.000) with a linear fitting. For fast progressing patients, there was also a change in global QoL the more they were physically impaired with a quadratic curve fitting. Patients with slow progression showed no significant changes in depressiveness or QoL the more they were physically disabled (Table 4).

**Table 4:** Subgroup analyses according to progression rate and time since symptom onset

Subgroups		Clinical data		Measures of wellbeing		
				Global QoL (ACSA)	Subjective QoL (SEIQoL)	Depressiveness (ADI-12)
Progression	Fast (≥ 1.4)	Physical disability (ALSFRS-r)	Curve fitting	Quadratic	No relation	Linear
			*R*^*2*^	.040		.056
			*p*	.010*		.000*
	Slow (< 1.4)		Curve fitting	No relation	No relation	No relation
			*R*^*2*^			
			*p*			
Time since onset	Long (> 60 months)		Curve fitting	Quadratic	No relation	No relation
			*R*^*2*^	.137		
			*p*	.017*		
	Short (≤ 60 months)		Curve fitting	Linear	No relation	Linear
			*R*^*2*^	.087		.052
			*p*	.000*		.000*

Regression analysis with curve fitting for subgroups according to progression rate (“fast progressors” with ≥ 1.4 and “slow progressors” with < 1.4 loss of points on the ALSFRS-r per month) and time since symptom onset (> 60 and ≤ 60 months since symptom onset); always the best fit is displayed

*Significance with level of *p* < 0.05

For long surviving patients (> 60 months since onset), there was a significant association of physical function decline and global QoL with a quadratic curve fitting, indicating a drop in QoL in patients with mild to moderate physical impairments (indicated by a high ALSFRS-r score) and an increase in global QoL for those long surviving patients with more severe physical restrictions. There was no significant relation between physical disability and subjective QoL or depressiveness in long surviving patients.

For those patients with shorter time with the disease (≤ 60 months since onset), there is a linear curve fitting for physical disability (indicated by a high ALSFRS-r score) and both global QoL and depressiveness. This demonstrates that patients with a short time since onset and a low state of physical functions have a linearly reduced wellbeing (high depressiveness and low global QoL), the lower the state of physical disabilities (Table 4).

## Discussion

Being confronted with the fatal diagnosis of ALS is a major challenge for the patients and their families. It has been suggested that after the first shock of being confronted with the fatal diagnosis and in the early stages of the disease, ALS patients might develop a low wellbeing indicated by depressive symptoms and loss of QoL [8, 14]. Triggering factors of depressiveness might be multiple psychological and social issues associated to loss of health and autonomy, and the end of life itself [23, 24]. At this time, patients need to cope with feelings of sadness and uncertainty [25, 26]. In line with this concept, some patients in our study showed increased depressiveness and decreased QoL at the time of the first interview, which was conducted after a short period of diagnosis communication in many patients. However, the majority of patients presented a rather satisfactory QoL, showed no depressiveness, and these parameters did not significantly change between the first and the second interview in line with previous studies [27]. Even though the diagnosis of ALS is devastating and the psychological wellbeing of the patient decreases at first, in a longer period of time patients might adapt their expectations to the actual circumstances according to the TOTE model [28] and finally accept their condition, finding the contentment in life again, so psychological wellbeing can remain stable. In accordance, our longitudinal assessment of psychological wellbeing in ALS suggested a stability of some measures of wellbeing within a period of 12 months, including both global and subjective QoL. This was true despite decrease in physical function following the natural course of the disease, demonstrating that patients affected with ALS for longer periods of time might habituate to the situation. These results are consistent with those of Matuz et al. [12], suggesting that patients can eventually adjust to the situation over time, possibly through gradual adaptation processes due to successful coping strategies [2, 10, 12, 29, 30].

Only one fourth of the participating patients presented clinically relevant depressive symptoms at the initial measurement and after 12 months, nevertheless the mean scores for depressiveness in both measurements were under the threshold for clinically relevant depression, which is in line with earlier findings [30, 31]. Thus, depressiveness is not necessarily an unavoidable consequence of ALS, but being present only in some patients. Contrary to other measures of wellbeing, depressiveness showed a slight increase within a year. In addition, within the regression analysis, depressiveness similar to global QoL were associated with physical function loss. These results do not follow the concept of lack of worsening during the course of the disease [8, 32], which might possibly be explained by different clinical characteristics in different clinical samples, as will be explained next. According to the quadratic curve shown in global QoL, patients who are not yet very physically impaired tend to have a good QoL. For those patients with an ALSFRS-r score of around 35 to 18 points, the QoL seems to be low, but for those patients with really impaired physical function, QoL tend to be high again. This indicates that subgroups of ALS-patients are able to cope well with the situation [12]. This was especially true for long surviving patients who live with the disease for 5 years and more, and can be regarded as the clinical exception to the rule (true for about 5–10% of patients). A majority of patients are expected to live up to 5 years [33], and within this sample (including “fast progressors” and those with short time after symptom onset), global QoL declines and depressiveness increases in the course of physical decline. This is in line with previous studies, showing that depression is associated with increase in physical impairment in these subsamples [8, 9, 32]. If patients pass the point of expected time of survival (5 years or more), patients have to realize that living with this disease is not simply a transient state to be passed before death eventually comes, but that living with physical impairments has to be incorporated into daily life. These patients might produce bias in empirical studies and clinical trials, as they are more motivated and are more likely to participate in studies.

In the current study, 20% of patients had a fast progression rate, and the cubic and logarithmic association of progression and global QoL or depressiveness, respectively, highlights the fact that progression rate plays an important role in the development of depressive symptoms in ALS. The logarithmic curve fit for disease progression and depressiveness indicates that patients show little signs of depressive symptoms when the disease progresses slowly. Lulé et al. [2] stated that patients with fast progression have no time to adapt to the situation and to cope with the rapid changes in life associated with immobility and thus, they are more likely to suffer from depression. Therefore, the tendency to depressiveness stipulates that good psychosocial adjustment might not apply to some patients with fast progression or to those whose physical abilities are severely reduced when first attending the clinic (typically those being the “fast progressors”). Interestingly, even in the group of fast progressors, there was a quadratic curve fit of global QoL and physical function loss, indicating that even in a subsample of patients with a fast progressing disease, QoL can nevertheless stay high.

Opposed to global QoL and depressiveness, patients’ subjective QoL was not associated with their physical disabilities or progression rate. This confirms the definition that subjective QoL comprises personal priorities and other important aspects of life that are not necessarily health related [34, 35]. Patients might adjust these preferences and expectations over time, and focus on the aspects that are not affiliated to physical disabilities [36]. For instance, patients with fatal illness might re-evaluate their priorities regarding soft values, such as positive contact to family and friends, as the most important factors in life to find meaning and hope in living [13, 27, 34, 37, 38].

### Limitations and recommendations

This study may be biased because some patients declined to participate in the study possibly due to lack of motivation, which could be especially true for depressive patients. However, as only a minority of 47 patients declined participation due to unwillingness, without any clinical evidence of depressiveness, this effect might be minor. Further, wellbeing is a highly complex concept, which encompasses a multitude of facets. Measuring QoL and depressiveness are only some possible measures to determine wellbeing, but still might not provide a full picture of what is actually on the patient’s mind. In addition, patients might tend to provide expected answers and might not be ready to tell everything they think or feel. This might be shared only with the closest relatives. Further, the complexity of patient’s personality can hardly be squeezed into the tight concept of some standardized questionnaires. Thus, it can be in no way expected to fully explain the patient’s actual emotional state. All this might be true for this and most empirical studies on wellbeing, but instead of generally discarding any such work, this type of research can be regarded as another flash of a torch on the picture of what living with ALS actually means.

Future research would be helpful in continuing to discover how to maintain the wellbeing of ALS patients. We know from previous studies how much patient’s wellbeing is interrelated with caregiver’s wellbeing [39, 40]. A longitudinal evaluation of the dyad of primary caregiver’s and patient’s wellbeing would be important to understand how caregiver’s wellbeing is affected by patient’s disability and progression rate and how caregivers’ and patients’ wellbeing interfere. Future work should help to further define key factors to maintain a good QoL to allow for patient centered medical counseling and possible psychological interventions for both parties. Furthermore, an assessment of the psychoactive medications prescribed to the patients, compliance with medication and impact of medication on patient’s actual wellbeing (including QoL) would also be interesting to address in future studies.

## Conclusion

The first months after the diagnosis of ALS might be a crisis-loaded period when patients need special attention in clinical counseling. However, after the possible shock caused by the devastating news, many patients adapt to the new circumstances and have a rather stable subjective wellbeing over time. Especially long surviving patients, who have time to adapt to the situation and change their expectations, maintain a good QoL and show no depressive symptoms. This underlines that despite the fatal diagnosis, especially long surviving ALS patients may still value life and show no general end-of-life-oriented despair [11, 14, 41]. These patients rarely attend the clinics and might not be considered in the concept of clinical counseling. Our results also suggest that increased physical disability and fast progression rate might predict lower psychological wellbeing in ALS with a higher risk of dissatisfaction, depressiveness or even wish for ending life after the diagnosis. These clinical parameters (e.g., fast progression and low physical function when first attending the clinics) might need special attention in the sense of red flags in clinical counseling.

We hereby present conclusive evidence that the dynamics of wellbeing following the diagnosis shows multiple facets, mostly depending on different clinical parameters. Any clinician with experience in the field of ALS knows how difficult it is to predict the patient’s way to cope with the disease [42]. With this work, we provide some key elements, which determine possible dynamics of wellbeing following diagnosis.

## Data Availability

The anonymous demographics and data will be shared on request from first and corresponding author.

## References

[1] Kiernan MC (2012). Amyotrophic lateral sclerosis and frontotemporal dementia. J Neurol Neurosurg Psychiatry.

[2] Lulé D, Ehlich B, Lang D, Sorg S, Heimrath J, Kübler A, Birbaumer N, Ludolph AC (2013). Quality of life in fatal disease: the flawed judgement of the social environment. J Neurol.

[3] Simmons Z, Bremer BA, Robbins RA, Walsh SM, Fischer S (2000). Quality of life in ALS depends on factors other than strength and physical function. Neurology.

[4] Kübler A, Winter S, Ludolph AC, Hautzinger M, Birbaumer N (2005). Severity of depressive symptoms and quality of life in patients with amyotrophic lateral sclerosis. Neurorehabil Neural Repair.

[5] Shamshiri H, Fatehi F, Abolfazli R, Harirchian MH, Sedighi B, Zamani B, Roudbari A, Razazian N, Khamseh F, Nafissi S (2016). Trends of quality of life changes in amyotrophic lateral sclerosis patients. J Neurol Sci.

[6] Hillemacher T, Grässel E, Tigges S, Bleich S, Neundörfer B, Kornhuber J, Hecht MJ (2004). Depression and bulbar involvement in amyotrophic lateral sclerosis. Amyotroph Lateral Scler Other Motor Neuron Disord.

[7] Cragg JJ, Seals R, Cashman N, Weisskopf MG (2016). Journal club: depression before and after diagnosis with amyotrophic lateral sclerosis. Neurology.

[8] Körner S, Kollewe K, Abdulla S, Zapf A, Dengler R, Petri S (2015). Interaction of physical function, quality of life and depression in amyotrophic lateral sclerosis: characterization of a large patient cohort. BMC Neurol.

[9] Rabkin JG, Goetz R, Factor-Litvak P, Hupf J, McElhiney M, Singleton J, Mitsumoto H (2015). Depression and wish to die in a multicenter cohort of ALS patients. Amyotroph Lateral Scler Frontotemporal Degener.

[10] Lulé D, Zickler C, Häcker S, Bruno MA, Demertzi A, Pellas F, Laureys S, Kübler A (2009). Life can be worth living in locked-in syndrome. ProgBrain Res.

[11] Kuzma-Kozakiewicz M, Andersen PM, Ciecwierska K, Vázquez C, Helczyk O, Loose M, Uttner I, Ludolph AC, Lulé D (2019). An observational study on quality of life and preferences to sustain life in locked-in state. Neurology.

[12] Matuz T, Birbaumer N, Hautzinger M, Kübler A (2015). Psychosocial adjustment to ALS: a longitudinal study. Front Psychol.

[13] Lulé D, Pauli S, Altintas E, Singer U, Merk T, Uttner I, Birbaumer N, Ludolph AC (2012). Emotional adjustment in amyotrophic lateral sclerosis (ALS). J Neurol.

[14] Lulé D, Nonnenmacher S, Sorg S, Heimrath J, Hautzinger M, Meyer TD, Kübler A, Birbaumer N, Ludolph AC (2014). Live and let die: existential decision processes in a fatal disease. J Neurol.

[15] Ludolph AC, Drory V, Hardiman O, Nakano I, Ravits J, Robberecht W, Shefner J (2015). A revision of the El Escorial criteria: 2015. Amyotroph Lateral Scler Frontotemporal Degener.

[16] Strong MJ, Abrahams S, Goldstein LH, Woolley S, Mclaughlin P, Snowden J, Mioshi E, Roberts-South A, Benatar M, HortobáGyi T, Rosenfeld J, Silani V, Ince PG, Turner MR (2017). Amyotrophic lateral sclerosis - frontotemporal spectrum disorder (ALS-FTSD): Revised diagnostic criteria. Amyotroph Lateral Scler Frontotemporal Degener.

[17] Cedarbaum JM, Stambler N, Malta E, Fuller C, Hilt D, Thurmond B, Nakanishi A (1999). The ALSFRS-R: a revised ALS functional rating scale that incorporates assessments of respiratory function. J Neurol Sci.

[18] Kollewe K, Mauss U, Krampfl K, Petri S, Dengler R, Mohammadi B (2008). ALSFRS-R score and its ratio: a useful predictor for ALS-progression. J Neurol Sci.

[19] Hammer EM, Häcker S, Hautzinger M, Meyer TD, Kübler A (2008). Validity of the ALS-Depression-Inventory (ADI-12)–a new screening instrument for depressive disorders in patients with amyotrophic lateral sclerosis. J Affect Disord.

[20] Bernheim JL, Buyse M (1993). The Anamnestic Comparative Self-Assessment for Measuring the Subjective Quality of Life of Cancer Patients. J Psychosoc Oncol.

[21] Hickey AM, Bury G, O’Boyle CA, Bradley F, O'Kelly FD, Shannon W (1996). A new short form individual quality of life measure (SEIQoL-DW): application in a cohort of individuals with HIV/AIDS. BMJ.

[22] Andersen PM, Kuzma-Kozakiewicz M, Keller J, Aho-Özhan HEA, Ciecwierska K, Szejko N, Vázquez C, Böhm S, Badura-Lotter G, Meyer TD, Petri S, Linse K, Hermann A, Semb O, Stenberg E, Nackberg S, Dorst J, Uttner I, Häggström A-C, Ludolph AC, Lulé D (2018). Therapeutic decisions in ALS patients: cross-cultural differences and clinical implications. J Neurol.

[23] McLeod JE, Clarke DM (2007). A review of psychosocial aspects of motor neurone disease. J Neurol Sci.

[24] Caga J, Ramsey E, Hogden A, Mioshi E, Kiernan MC (2015). A longer diagnostic interval is a risk for depression in amyotrophic lateral sclerosis. Palliat Support Care.

[25] Taylor L, Wicks P, Leigh PN, Goldstein LH (2010). Prevalence of depression in amyotrophic lateral sclerosis and other motor disorders. Eur J Neurol.

[26] Real RGL, Herbert C, Kotchoubey B, Wessig C, Volkmann J, Kübler A (2014). Psychophysiological correlates of coping and quality of life in patients with ALS. Clin Neurophysiol.

[27] Jakobsson Larsson B, Ozanne AG, Nordin K, Nygren I (2017). A prospective study of quality of life in amyotrophic lateral sclerosis patients. Acta Neurol Scand.

[28] Miller GA, Galanter E, Pribram KH (1960). Plans and the structure of behavior.

[29] Montel S, Albertini L, Desnuelle C, Spitz E (2012). Evolution of quality of life, mental health, and coping strategies in amyotrophic lateral sclerosis: a pilot study. J Palliat Med.

[30] Lulé D, Häcker S, Ludolph AC, Birbaumer N, Kübler A (2008). Depression and quality of life in patients with amyotrophic lateral sclerosis. Dtsch Arztebl Int.

[31] Grehl T, Rupp M, Budde P, Tegenthoff M, Fangerau H (2011). Depression and QOL in patients with ALS: how do self-ratings and ratings by relatives differ?. Qual Life Res.

[32] Thakore NJ, Pioro EP (2016). Depression in ALS in a large self-reporting cohort. Neurology.

[33] Andersen PM, Abrahams S, Borasio GD, de Carvalho M, Chio A, van Damme P, Hardiman O, Kollewe K, Morrison KE, Petri S, Pradat P-F, Silani V, Tomik B, Wasner M, Weber M (2012). EFNS guidelines on the clinical management of amyotrophic lateral sclerosis (MALS)–revised report of an EFNS task force. Eur J Neurol.

[34] Clarke S, Hickey AM, O'Boyle CA, Hardiman O (2001). Assessing individual quality of life in amyotrophic lateral sclerosis. Qual Life Res.

[35] Matuz T, Birbaumer N, Hautzinger M, Kübler A (2010). Coping with amyotrophic lateral sclerosis: an integrative view. J Neurol Neurosurg Psychiatry.

[36] Nordeson A, Engström B, Norberg A (1998). Self-reported quality of life for patients with progressive neurological diseases. Qual Life Res.

[37] Neudert C, Wasner M, Borasio GD (2001). Patients' assessment of quality of life instruments: a randomised study of SIP, SF-36 and SEIQoL-DW in patients with amyotrophic lateral sclerosis. J Neurol Sci.

[38] Neudert C, Wasner M, Borasio GD (2004). Individual quality of life is not correlated with health-related quality of life or physical function in patients with amyotrophic lateral sclerosis. J Palliat Med.

[39] Galvin M, Gaffney R, Corr B, Mays I, Hardiman O (2017). From first symptoms to diagnosis of amyotrophic lateral sclerosis: perspectives of an Irish informal caregiver cohort-a thematic analysis. BMJ Open.

[40] Burke T, Hardiman O, Pinto-Grau M, Lonergan K, Heverin M, Tobin K, Staines A, Galvin M, Pender N (2018). Longitudinal predictors of caregiver burden in amyotrophic lateral sclerosis: a population-based cohort of patient-caregiver dyads. J Neurol.

[41] Linse K, Rüger W, Joos M, Schmitz-Peiffer H, Storch A, Hermann A (2017). Eye-tracking-based assessment suggests preserved well-being in locked-in patients. Ann Neurol.

[42] Aho-Özhan HEA, Böhm S, Keller J, Dorst J, Uttner I, Ludolph AC, Lulé D (2017). Experience matters: neurologists’ perspectives on ALS patients’ well-being. J Neurol.

